# Exposure to family and domestic violence in the prenatal period is associated with an increased risk of hospitalization for bronchiolitis in children under 2 years

**DOI:** 10.1093/pubmed/fdae120

**Published:** 2024-06-26

**Authors:** Carol Orr, Erin Kelty, Patricia Belinelo, Colleen Fisher, A Rebecca Glauert, Melissa O’Donnell, David B Preen

**Affiliations:** School of Population and Global Health, The University of Western Australia, Perth, Western Australia 6009, Australia; School of Population and Global Health, The University of Western Australia, Perth, Western Australia 6009, Australia; The School of Medicine, University of Notre Dame, Fremantle, Western Australia 6160, Australia; School of Population and Global Health, The University of Western Australia, Perth, Western Australia 6009, Australia; School of Population and Global Health, The University of Western Australia, Perth, Western Australia 6009, Australia; Australian Centre for Child Protection, The University of South Australia, Adelaide, South Australia 5000, Australia; School of Population and Global Health, The University of Western Australia, Perth, Western Australia 6009, Australia

**Keywords:** children, epidemiology, violence

## Abstract

**Background:**

Existing research has acknowledged a correlation between stress in pregnancy and poorer respiratory health in offspring. However, research focusing on stress caused by family and domestic violence in the prenatal period is missing.

**Methods:**

A retrospective cohort study included children born 1987–2010 who were identified as being exposed to FDV in the prenatal period (*n* = 1477) from two sources: WA Police Information Management System and WA Hospital Morbidity Data Collection (HMDC) and a non-exposed comparison group (*n* = 41 996). Hospitalization for bronchiolitis was identified in HMDC. Cox regression was used to estimate the adjusted and unadjusted hazard ratio and 95% confidence interval for bronchiolitis hospitalizations contact.

**Results:**

Children exposed to FDV had a 70% (HR 1.70, 95% CI: 1.49–1.94) increased risk of hospitalization for bronchiolitis than non-exposed counterparts by age two. Children exposed to FDV had a longer average hospital stay for bronchiolitis than non-exposed children (4.0 days vs. 3.8 days, *P* < 0.001).

**Conclusions:**

Prenatal exposure to FDV is associated with bronchiolitis hospitalization in children <2 years. Along with other risk factors, clinicians should give consideration to maternal stress factors, including experiencing FDV as a potential contributor to bronchiolitis.

## Introduction

Family and domestic violence (FDV) is a public health crisis. FDV refers to acts and/or threats of violence or abuse, including physical, non-physical and/or sexual, between current or former intimate partners as well as family members^1^; the acts can be either criminal or non-criminal. The use of the term FDV should not detract from the fact that it is a gendered act that is disproportionately perpetrated by men against women.^2^ Within Australia 1 in 4 women will experience FDV in their lifetime.^3^ While there is conflicting evidence, many studies report that pregnancy is associated with an increased risk for FDV,^4^^,^^5^ and that FDV during pregnancy is known to increase psychological stress for the mother.^6^^,^^7^

Stress during pregnancy can result in dysregulation of the hypothalamic-pituitary-adrenal (HPA) axis activity and elevation of cortisol, which is thought to directly affect foetal development.^8^ This dysregulation can predispose a child to poorer lung function and respiratory infections in early life^9^^,^^10^ and increases the risk of developing asthma and chronic obstructive pulmonary disease (COPD) later in life.^11–13^ Furthermore, stress during pregnancy is associated with changes in immune system functioning, including increased levels of inflammatory markers, such as interleukin-6 (IL-6) and C-reactive protein (CRP).^14^ The high levels of inflammatory markers during pregnancy are increasingly recognized as a risk factor for adverse pregnancy and birth outcomes^11^^,^^14^^,^^15^ and may influence offspring development.^16^^,^^17^

A recent longitudinal study highlighted that children exposed to FDV in the prenatal period were more likely to be hospitalized for a range of health issues when compared to non-exposed children, including respiratory and infectious disorders.^18^ Bronchiolitis is one of the most common lower respiratory infections that affect young children, predominantly those under 2 years of age.^19^ It is a seasonal illness, peaking during autumn and winter^20^, and in most instances, a mild, self-limiting infection in children that can be treated in the community.^21^ As such, it is regarded as an illness where hospitalizations are potentially preventable.^22^ Despite this, bronchiolitis is the most common cause of respiratory hospitalization in children under 2 years of age.^23^ Children who are at particular risk of bronchiolitis include those born pre-term and Aboriginal children.^20^ Within Australia, Aboriginal people are more likely to have poorer health than their non-Aboriginal counterparts. This discrepancy has been acknowledged in government policy, and there has been a push to gain more understanding of the disparity to support closing this gap.

Our aim was to examine hospitalizations for bronchiolitis in children exposed to FDV in the prenatal period compared to peers who were not exposed in the prenatal period. It is hypothesized that exposure to FDV in the prenatal period will be associated with an increased risk of hospitalization for bronchiolitis in children aged <2 years.

## Methods

### Study design

This study was a retrospective longitudinal cohort study using linked administrative health and social sector datasets, linked by the Western Australia (WA) Data Linkage Branch. The data collections were linked, at the individual level, by the WA Data Linkage Branch using probabilistic matching with clerical review.^24^ The process of linkage and clerical review used by the WA Data Linkage Branch has been shown to be 99.89% accurate.^24^ De-identified data with encrypted project-specific identifications for each member of the cohort and their mother were supplied to the researchers, allowing the datasets to be merged.

### Study cohort

**Fig. 1:**
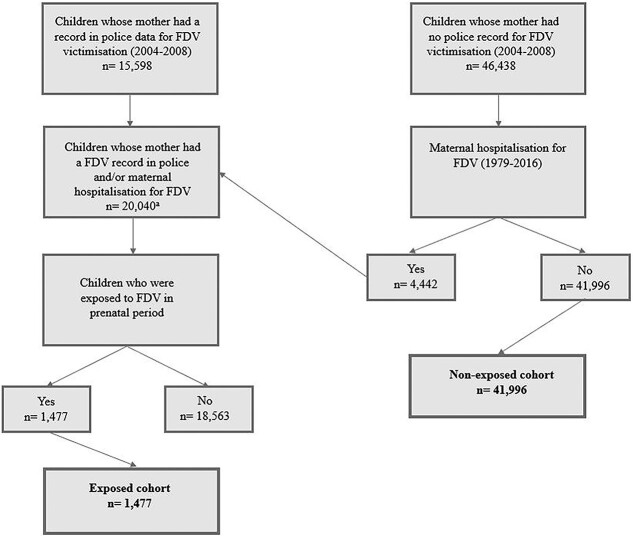
Cohort flowchart includes the 4442 children identified in the original cohort of children whose mother had no police record for FDV victimization (2004–2008) but had a maternal hospitalization for FDV.

The study included 1477 children born from 1987 to 2010 to a mother who was a victim of FDV 12 months prior to the child’s birth (prenatal period) and a non-FDV-exposed comparison group of 41 996 children. The cohort is derived from a larger study population originally matched 1:3 (exposed:non-exposed) on sex, month of birth, Aboriginal status and socioeconomic status (SES) (Fig. 1).

### Identification of FDV exposure

FDV was first identified in the WA Police Information Management System (IMS) (2004–2008), where the child’s mother was a victim and a male perpetrator was charged with an offence. The offence categories were derived using the Australian and New Zealand Standard Offence Classification subdivision level^25^ and comprised of murder, attempted murder, physical assault, sexual assault, threatening behaviour and misuse of weapons. WA Police Force data have a mandatory ‘domestic flag’ variable, which has been recorded since the implementation of the Frontline IMS system in 2004. This variable draws on the type of behaviour alleged and the relationship between the parties. Second, due to the hidden nature of FDV, maternal Hospital Morbidity Data Collection (HMDC) records (1979–2016) were interrogated for FDV-related hospitalizations in both the original exposed and non-exposed cohorts (Fig. 1), using International Classification of Disease (ICD) codes used in previous research.^26^ The HMDC contains records of hospital separations from all private and public hospitals within WA.

### Characteristics

Mother/child relationship was identified through the WA Family Connections System.^27^ Neighbourhood-level SES was determined by the Socio-Economic Indexes for Areas (SEIFA)^28^ using the WA Midwives Notification System (MNS) data (1987–2010), which contains data for all babies born >400 g in weight or of at least 20 weeks gestation. Quintiles of disadvantage were assigned to National Census collection districts (~250 households), ranging from 1 (high disadvantage) to 5 (low disadvantage).^28^ Residential remoteness was identified in the MNS data from collection district, the smallest spatial unit available^29^ and categorized as major cities, inner regional, outer regional, remote and very remote. The MNS also provided data on the child’s sex, gestational age, mother’s age at child’s birth, father’s age at child’s birth and mother’s marital status. Date of death for any children dying during the study period was determined from the WA Death Register (1987–2015). Child disability was obtained from the WA Register of Developmental Anomalies (1987–2015) and the Intellectual Disability Exploring Answers (IDEA) database (1987–2015). The WA Data Linkage Branch-derived Aboriginal status flag was used to identify Aboriginal children.^30^ Data for all sociodemographic and clinical variables used were complete apart from father’s age; therefore, we created a missing category in the fathers age variable.

### Outcome ascertainment: bronchiolitis

Within the HMDC data, each separation is coded with a single primary diagnosis and up to 20 co-diagnoses. To identify bronchiolitis hospitalizations in the first 2 years of life, HMDC records were interrogated from 1987 to 2012 (end of follow-up) using the Tenth Revision ICD with Australian modifications (ICD-10-AM) code J21. The ICD-10-AM code J21 was mapped^31^ to the equivalent ninth revision of ICD with clinical modifications (ICD-9-CM) to capture hospitalizations prior to July 1999, ICD-9-CM code 466.1.

### Analysis

Descriptive statistics were performed for all cohort characteristics and outcome measures. Pearson’s chi-square test was used to initially assess crude differences between exposed and non-exposed children’s characteristic variables. Multivariable Cox regression was used to estimate the adjusted and unadjusted hazard ratio (HR) and 95% confidence interval (CI) for the first hospital contact for bronchiolitis, comparing FDV-exposed and non-exposed children. A model adjustment was made for sex, Aboriginal status, SES, residential remoteness, maternal and paternal age, mothers’ marital status, gestational age, disability and season of birth. Data were censored when the child turned 2 years, date of death, or if there was no bronchiolitis contact by the end of follow-up (when the child reached 2-years of age). Aboriginal children have been shown to be at greater risk of FDV exposure due to the higher rates of FDV in Aboriginal communities.^26^^,^^32^ Therefore, separate models were run that are stratified by Aboriginal status. The length of stay was calculated for each child by taking an average of all hospitalization days for bronchiolitis. All analyses were conducted using SAS^®^ statistical software.

### Ethics

Ethics approval for this study was obtained from the WA Department of Health Human Research Ethics Committee (#2016/60), the WA Aboriginal Health Ethics Committee (#756), and the University of Western Australia Human Research Ethics Committee (#RA/4/1/8867).

## Results

Table 1 displays the characteristics of the cohort stratified by FDV exposure. Compared to non-exposed peers, children exposed to FDV in the prenatal period had a greater likelihood of having a hospitalization for bronchiolitis (16.2% vs. 7.8%, *P* < 0.0001), being Aboriginal (64.9% vs. 49.8%, *P* < 0.0001), being born to a mother who had never married (31.8% vs. 18.9%, *P* < 0.0001) and born prematurely (16.7% vs. 10.4%, *P* < 0.0001).

**Table 1:** Cohort clinical and sociodemographic characteristics

Characteristic		Childhood FDV	No recorded childhood FDV
		*N* (%)	*N* (%)
Aboriginal^*^			
	Yes	958 (64.9)	20 919 (49.8)
	No	519 (35.1)	21 077 (50.2)
Sex			
	Female	740 (50.1)	20 537 (48.9)
	Male	737 (49.9)	21 459 (51.1)
Born prior to 37 weeks gestation^*^			
	Yes	247 (16.7)	4353 (10.4)
	No	1230 (83.3)	37 643 (89.6)
Mother age at birth^*^			
	<20 years	218 (14.8)	6122 (14.6)
	20–29 years	864 (58.5)	22 774 (54.2)
	30–39 years	378 (25.6)	12 478 (29.7)
	40+ years	17 (1.2)	622 (1.5)
Fathers age at birth^*^			
	<20 years	68 (4.6)	2260 (5.4)
	20–29 years	514 (34.8)	16 754 (39.9)
	30–39 years	340 (23.0)	14 703 (35.0)
	40+ years	83 (5.6)	2829 (6.7)
	Age missing	472 (32.0)	5450 (13.0)
Maternal marital status at birth^*^			
	Married/de facto/widowed	932 (63.1)	33 300 (79.3)
	Never married	469 (31.8)	7920 (18.9)
	Divorced/separated	58 (3.9)	564 (1.3)
	Unknown/not stated	18 (1.2)	212 (0.5)
Socioeconomic status^*^			
	1-Most disadvantaged	792 (53.6)	20 569 (49.0)
	2	294 (19.9)	9549 (22.7)
	3	201 (13.6)	6251 (14.9)
	4	130 (8.8)	3867 (9.2)
	5-Least disadvantaged	60 (4.1)	1760 (4.2)
Residential remoteness^*^			
	Major cities	631 (42.7)	20 261 (48.2)
	Inner regional	144 (9.7)	4520 (10.8)
	Outer regional	225 (15.2)	6725 (16.0)
	Remote	190 (12.9)	5190 (12.4)
	Very remote	287 (19.4)	5300 (12.6)
Season of birth			
	Summer	372 (25.2)	10 297 (24.5)
	Autumn	373 (25.3)	10 827 (25.8)
	Winter	350 (23.7)	10 689 (25.5)
	Spring	382 (25.7)	10 183 (24.2)
Disability			
	Yes	145 (9.8)	3061 (7.3)
	No	1332 (90.2)	38 935 (92.7)
Hospitalization for bronchiolitis 0–2 years^*^			
	Yes^a^	239 (16.2)	3270 (7.8)
	No	1238 (83.8)	38,726 (92.2)

^*^Denotes that *P*-value is <0.0001for all characteristics between exposed and non-exposed children; SD = standard deviation.

Before adjustment for clinical and sociodemographic characteristics (Table 2), children exposed to FDV in the prenatal period had a 121% increased risk of being hospitalized for bronchiolitis when compared to non-exposed peers (HR = 2.21, 95% CI: 1.93–2.52). This risk attenuated, but remained significant, when model adjustments were made (aHR = 1.70, 95% CI: 1.49–1.94) (Table 2).

**Table 2:** Risk of hospitalization for bronchiolitis in all children aged 0–2 years

Characteristic		Crude HR (95% CI)	Adjusted^*^ HR (95%CI)
Sex			
	Male	1.35 (1.26–1.44)	1.33 (1.25–1.43)
	Female	Reference group	Reference group
Aboriginal status			
	Yes	3.53 (3.26–3.82)	2.62 (2.39–2.87)
	No	Reference group	Reference group
Socioeconomic status			
	1-Most disadvantaged	2.11 (1.71–2.61)	1.23 (0.99–1.53)
	2	1.43 (1.14–1.78)	1.06 (0.85–1.33)
	3	1.32 (1.05–1.66)	1.08 (0.86–1.36)
	4	1.05 (0.82–1.34)	0.90 (0.70–1.15)
	5-Least disadvantaged	Reference group	Reference group
Residential remoteness			
	1-Highly accessible	Reference group	Reference group
	2	0.97 (0.86–1.10)	0.98 (0.86–1.12)
	3	1.27 (1.15–1.40)	1.00 (0.90–1.10)
	4	1.56 (1.41–1.73)	1.04 (0.94–1.16)
	5-Very remote	2.30 (2.11–2.51)	1.22 (1.11–1.34)
Mother’s marital status			
	Married/defacto/widowed	Reference group	Reference group
	Never married	1.82 (1.69–1.96)	1.14 (1.05–1.24)
	Divorced/separated	1.72 (1.36–2.18)	1.29 (1.02–1.64)
	Unknown	2.13 (1.51–3.01)	1.28 (0.90–1.80)
Gestation			
	<37 weeks	2.60 (2.40–2.82)	2.10 (1.94–2.29)
	37 weeks+	Reference group	Reference group
Mothers age group			
	<20	2.30 (2.08–2.55)	1.23 (1.08–1.40)
	20–29	1.55 (1.43–1.69)	1.17 (1.06–1.29)
	30–39	Reference group	Reference group
	40+	1.30 (0.96–1.76)	1.28 (0.94–1.74)
Father’s age group			
	<20	2.20 (1.92–2.52)	1.10 (0.93–1.29)
	20–29	1.49 (1.37–1.62)	1.05 (0.95–1.16)
	30–39	Reference group	Reference group
	40+	0.98 (0.83–1.17)	0.97 (0.81–1.15)
	Missing	2.80 (2.54–3.08)	1.28 (1.15–1.43)
Season of birth			
	Summer	Reference group	Reference group
	Autumn	1.25 (1.14–1.37)	1.24 (1.13–1.36)
	Winter	1.16 (1.06–1.27)	1.15 (1.04–1.26)
	Spring	0.85 (0.77–0.94)	0.86 (0.78–0.96)
Child disability			
	Yes	1.87 (1.69–2.07)	1.63 (1.47–1.81)
	No	Reference group	Reference group
FDV exposure			
	Yes	2.21 (1.93–2.52)	1.70 (1.49–1.94)
	No	Reference group	Reference group

^*^Adjusted for all characteristic variables in table.

When analysis was stratified by Aboriginal status (Table 3), Aboriginal children exposed to FDV in the prenatal period had a 66% greater risk of hospitalization for bronchiolitis than non-exposed Aboriginal children (aHR = 1.66, 95% CI: 1.43–1.92). For non-Aboriginal children exposed to FDV, an 84% increased risk of hospitalization for bronchiolitis was observed compared to non-exposed peers (aHR = 1.84, 95% CI: 1.34–2.54), when controlling for all factors.

**Table 3:** Risk of hospitalization for bronchiolitis stratified by Aboriginal status

Characteristic		Aboriginal children adjusted^*^ HR (95% CI)	Non-Aboriginal children adjusted^*^ HR (95% CI)
Sex			
	Male	1.31 (1.21–1.41)	1.42 (1.23–1.64)
	Female	Reference group	Reference group
Socioeconomic status			
	1-Most disadvantaged	1.08 (0.82–1.43)	1.55 (1.10–2.19)
	2	0.97 (0.73–1.30)	1.17 (0.82–1.67)
	3	1.00 (0.74–1.36)	1.16 (0.81–1.67)
	4	0.87 (0.63–1.20)	0.92 (0.62–1.37)
	5-Least disadvantaged	Reference group	Reference group
Residential remoteness			
	1-Highly accessible	Reference group	Reference group
	2	1.04 (0.88–1.22)	0.88 (0.71–1.08)
	3	1.00 (0.89–1.13)	0.99 (0.81–1.20)
	4	1.07 (0.95–1.19)	0.85 (0.61–1.18)
	5-Very remote	1.27 (1.15–1.41)	0.62 (0.33–1.16)
Mother’s marital status			
	Married/defacto/widowed	Reference group	Reference group
	Never married	1.14 (1.04–1.24)	1.18 (0.93–1.49)
	Divorced/separated	1.18 (0.90–1.55)	1.81 (1.09–3.00)
	Unknown	1.28 (0.89–1.83)	1.23 (0.39–3.84)
Gestation			
	<37 weeks	2.00 (1.83–2.20)	2.59 (2.16–3.12)
	37 weeks+	Reference group	Reference group
Mothers age group			
	<20	1.21 (1.05–1.40)	1.37 (0.99–1.91)
	20–29	1.17 (1.04–1.32)	1.14 (0.96–1.36)
	30–39	Reference group	Reference group
	40+	1.50 (1.03–2.18)	0.94 (0.54–1.63)
Father’s age group			
	<20	1.08 (0.91–1.29)	1.24 (0.77–2.01)
	20–29	1.04 (0.92–1.17)	1.04 (0.87–1.24)
	30–39	Reference group	Reference group
	40+	0.97 (0.77–1.22)	0.94 (0.72–1.24)
	Missing	1.29 (1.13–1.46)	1.12 (0.79–1.59)
Season of birth			
	Summer	Reference group	Reference group
	Autumn	1.21 (1.09–1.34)	1.33 (1.10–1.61)
	Winter	1.16 (1.04–1.28)	1.10 (0.91–1.35)
	Spring	0.87 (0.77–0.98)	0.85 (0.68–1.04)
Child disability			
	Yes	1.51 (1.34–1.70)	2.05 (1.69–2.50)
	No	Reference group	Reference group
FDV exposure			
	Yes	1.66 (1.43–1.92)	1.84 (1.34–2.54)
	No	Reference group	Reference group

^*^Adjusted for all characteristic variables in table.

Children exposed to FDV who were hospitalized for bronchiolitis had a slightly longer stay than non-exposed children (4.0 days vs. 3.8 days, respectively, *P* < 0.0001). When stratified by Aboriginal status, Aboriginal children who were exposed to FDV stayed on average longer than non-exposed Aboriginal children (4.3 days vs. 3.9 days, *P* < 0.0001). Conversely, non-Aboriginal children exposed to FDV had a shorter length of stay than their non-exposed peers (2.7 vs. 3.2 days, *P* < 0.0001).

## Discussion

### Main findings of this study

In our study, exposure to FDV in the prenatal period was associated with an increased risk of hospitalization for bronchiolitis in children <2 years, even after adjustment for demographic and clinical characteristics.

### What is already known on this topic

Past research has highlighted that maternal stress during pregnancy is associated with an increased risk of respiratory infections in childhood,^33^ including bronchiolitis. The existing literature on prenatal stress exposure and bronchiolitis has predominantly focused on psychological stressors, such as anxiety^33^, or grouping multiple stressors, such as family problems, money worries and healthcare.^34^

### What this study adds

The current study extends existing knowledge on the type of prenatal stressor associated with bronchiolitis in early life, such as those outlined above, noting that FDV exposure in the prenatal period is significantly correlated with an increased risk of hospitalization for bronchiolitis.

The study further advances the understanding of the impact exposure to FDV in the prenatal period has on children’s health, specifically hospitalizations for bronchiolitis in children <2 years.

It is likely that stress plays an important role in the association between prenatal FDV and bronchiolitis in children <2 years. Future research is needed to gain a deeper understanding of the pathway, research should give consideration to biological samples, including biomarkers of inflammatory markers, HPA activity or epigenetic measures.

Aboriginal children exposed to FDV had, on average, longer hospitalizations for bronchiolitis than non-exposed Aboriginal children. Conversely, non-Aboriginal children exposed to FDV had shorter length of stays than non-exposed non-Aboriginal children. Our finding that Aboriginal children exposed to FDV have a longer hospital stay has some accord with previous research that found longer hospital stays for epilepsy in Aboriginal children exposed to FDV.^35^ One possible explanation is that a higher proportion of Aboriginal children exposed to FDV were from very remote areas. Remote locations are reliant on small health centres where clinicians are often generalists; the lack of specialist service availability may hinder discharge.^35^ However, regression models controlled for residential remoteness, thereby reducing this as the likely primary explanation for the observed difference. From the available data, it is difficult to draw a conclusion as to why non-Aboriginal children exposed to FDV had a shorter stay, on average than non-exposed, non-Aboriginal children; further research is required to investigate discrepancies in length of stay.

Aboriginal children were overrepresented in our cohort. Our exposed cohort comprised of 65% Aboriginal children compared to 7% observed in the WA child population (0–18) years.^36^ This may be, in part, explained by the higher rates of FDV in Aboriginal communities.^26^^,^^32^ A further possible explanation is that the FDV identified in police data required a charge to be made; there is evidence to suggest that Aboriginal Australians are more likely to be charged for a crime than non-Aboriginal Australians.^37^^,^^38^ Importantly, it is important to note that FDV is not consistent with traditional Aboriginal culture, and the overrepresentation of Aboriginal children in this study needs to be viewed in context of the multiple disadvantages faced by Aboriginal peoples as a result of colonization and not as a racial determinant.^39^^,^^40^

It is recognized that antenatal care provides an opportunity for clinicians to enquire about FDV^41^ due to its negative health impacts on both the mother and the fetus^42^ and because the course of the pregnancy provides an opportunity for the clinician to establish sufficient trust with the woman to facilitate disclosure.^43^ However, like many countries, screening for FDV in the prenatal period is not mandatory in Australia. It has been argued that there is insufficient evidence of the effectiveness of universal screening^44^^,^^45^, including improved outcomes for women or a reduction in the violence being experienced^46^ as a result of screening to warrant it. Despite an abundance of screening tools for FDV generally, this insufficient evidence may, in part, be attributed to the absence of a screening tool specifically for pregnant women^44^ and limited research examining health and social outcomes for women and their children following screening.^46^ In the absence of the former, tools used more broadly in screening have been tested for their accuracy in identifying FDV in the prenatal period. Zapata-Calvente and colleagues^47^ found that the WAST-Short is effective in accurately identifying FDV during pregnancy and could be used during prenatal visits.

Our findings provide further evidence for the importance of screening for FDV during the prenatal period, to mitigate the negative health impacts of FDV on both the mother and child. Further research into the identification of appropriate tools for screening during this period or the development of a specific tool, along with ensuring that identified women are provided appropriate support, is warranted.

The hospitalization for bronchiolitis provides another opportunity for intervention for children exposed to FDV. For many women, FDV continues for several years.^48^ It is important that clinicians take account of wider family circumstances, including FDV, when assessing the health and wellbeing of the child. To approach the issue of FDV safely, clinicians need to have skills and be competent to approach the subject of FDV with families in their care.

### Limitations of this study

The main strength of our study is the use of linked population health data to identify our cohort, rather than specialist services, such as child protection or refugee services. While addressed where possible, our study is not without limitations. Our capture of FDV was limited to police records where a male perpetrator was charged and from hospitalization records. Therefore, it can be assumed that our data are an underestimation of FDV and are not generalizable to children whose mothers’ access different services or no services at all. Furthermore, our data are limited in the range of FDV behaviours, being heavily biased toward physical assaults as non-physical violence is less likely to result in a hospitalization or police record. Additionally, the timeframe for identification of FDV differed in the hospital records and the police records; it is likely that within the ‘non-exposed’ cohort there are those that have been exposed to FDV but not identified due to the data limitations, potentially biasing our results towards the null.

A further limitation of the study is that we do not have maternal smoking data available for the full cohort or any data on smoking in the family home. Previous research has highlighted a correlation between passive smoking in the family home and bronchiolitis.^49–51^ Additionally, we do not have data on whether the child was breastfed or not. It is acknowledged that being breastfed can reduce the risk of bronchiolitis.^52^

For many women, FDV continues for years,^48^ it may be that the FDV in pregnancy continued post-pregnancy. The continued FDV may have impacted the parenting capacity of the mother, including her ability to access primary care health services for the child.^53^ These factors may have an impact on children with bronchiolitis being hospitalized and are an area in need of further investigation.

## Conclusion

Prenatal exposure to FDV is associated with bronchiolitis hospitalization in children <2 years. The findings add to the small but growing body of literature attesting to the negative physical health impacts of *in utero*-exposure to FDV and provide further evidence for the need for health professionals to undertake appropriate enquiry into maternal experiences of FDV during the prenatal period. It is also incumbent that, following identification, women are provided with support appropriate for their situation and expressed needs in order to reduce maternal stress and the associated consequences. Along with other risk factors, clinicians should give consideration to maternal stress factors, including experiencing FDV as a potential contributor to bronchiolitis.

## Data Availability

The datasets generated and/or analysed during the current study are not publicly available due to the terms of the ethics approval granted by the Department of Health Western Australia Human Research Ethics Committee and data disclosure policies of the Data Providers. The datasets may be available from the Western Australia Data Linkage Branch at dataservices@health.wa.gov.au and subject to the approval from the Department of Health Western Australia Human Research Ethics Committee and relevant custodians.

## Notes

**Carol Orr**, Research Fellow

**Erin Kelty**, Research Fellow

**Patricia Belinelo**, Lecturer

**Colleen Fisher**, Head of School

**A. Rebecca Glauert**, Principal Research Fellow

**Melissa O'Donnell**, Deputy Director

**David B. Preen**, Chair of Public Health
